# Magnitude of postpartum hemorrhage and associated factors among women who gave birth in Ethiopia: a systematic review and meta-analysis

**DOI:** 10.1186/s12978-022-01498-4

**Published:** 2022-09-21

**Authors:** Tadesse Tolossa, Getahun Fetensa, Edgeit Abebe Zewde, Merga Besho, Tafese Dejene Jidha

**Affiliations:** 1 Department of Public Health, Institutes of Health Sciences, Wollega University, P.O.BOX: 395, Nekemte, Ethiopia; 2 Department of Nursing, School of Nursing and Midwifery, Wollega University, Nekemte, Ethiopia; 3 Department of Biomedical Science, College of Health Sciences, Debretabor University, Debretabor, Ethiopia; 4 Department of Midwifery, School of Nursing and Midwifery, Wollega University, Nekemte, Ethiopia; 5 College of Medicine and Health Sciences, Dire Dawa University, Dire Dawa, Ethiopia; 6 Department of Health Behavior and society, Faculty of public Health, Jimma medical center, Jimma University, Jimma, Ethiopia

**Keywords:** Postpartum hemorrhage, Associated systematic factors, Systematic review, Ethiopia

## Abstract

**Background:**

Postpartum hemorrhage (PPH) is the leading cause of maternal mortality and morbidity worldwide, particularly in resource-limited countries such as Ethiopia. Findings from a few studies were inconsistent and inconclusive. Therefore, this study aimed to estimates the pooled magnitude of PPH and factors associated with PPH among women who gave birth in Ethiopia.

**Methods:**

Electronic databases such as Medline, Pub Med, Cochrane library, the Web of Science, and Google Scholar were used to search for articles. The search period for articles was conducted from 15th August 2021 to 15th November 2021. Data were extracted using a standardized data extraction checklist and the analyses were conducted using Stata version 14. The Cochrane Q test statistic and *I*^2^ statistics were used to assessing heterogeneity. To estimate the pooled magnitude of postpartum hemorrhage, a random-effects model was fitted. Association between PPH and independent variables was reported in odds ratio (OR) with 95% confidence interval (CI). Visual assessment of publication bias was assessed using a funnel plot and Egger’s test was used to check the significant presence of publication bias.

**Results:**

A total of 876 studies were identified from several databases and nine studies fulfilled eligibility criteria and were included in the meta-analysis. The pooled magnitude of PPH in Ethiopia was 11.14% (95% CI 7.21, 15.07). The current meta-analysis revealed that lack of antenatal care follow-up (ANC) (OR = 6.52, 95% CI 2.87, 14.81), being multipara (OR = 1.88, 95% CI 1.25, 2.85), and having the previous history of PPH (OR = 7.59, 95% CI 1.88, 30.55) were found to be significantly associated with PPH.

**Conclusion:**

In Ethiopia the magnitude of PPH was high, and lack of ANC up follow-up, being multipara, and having a previous history of PPH were risk factors for postpartum hemorrhage. Thus, improving antenatal care follow-up is needed to decrease the magnitude of postpartum hemorrhage.

## Plain Language summary

Postpartum hemorrhage (PPH) is the leading cause of maternal mortality and morbidity worldwide, particularly in resource-limited countries such as Ethiopia. Findings from a few studies were inconsistent and inconclusive. Therefore, this study aimed to estimate the pooled magnitude of PPH and factors associated with PPH among women who gave birth in Ethiopia.

Electronic databases such as Medline, Pub Med, Cochrane library, the Web of Science, and Google Scholar were used to search for articles. Data were extracted using a standardized data extraction checklist and the analyses were conducted using Stata version 14. The Cochrane Q test statistic and *I*^2^ statistics were used to assessing heterogeneity. To estimate the pooled magnitude of postpartum hemorrhage, a random-effects model was fitted. Association between PPH and independent variables was reported in odds ratio (OR) with 95% confidence interval (CI).

The pooled magnitude of PPH in Ethiopia was 11.14% (95% CI 7.21, 15.07). The current meta-analysis revealed that lack of antenatal care follow-up (ANC) (OR = 6.52, 95% CI 2.87, 14.81), being multipara (OR = 1.88, 95% CI 1.25, 2.85), and having the previous history of PPH (OR = 7.59, 95% CI 1.88, 30.55) were found to be significantly associated with PPH.

In Ethiopia the magnitude of PPH was high, and lack of ANC follow-up, being multipara, and having the previous history of PPH were risk factors for postpartum hemorrhage. Thus, improving antenatal care follow-up is needed to decrease the magnitude of postpartum hemorrhage.

## Background

Postpartum hemorrhage (PPH) is defined as blood loss over 500 ml following vaginal deliveries and more than 1000 ml in cesarean section deliveries which have the potential to compromise the hemodynamic process [1, 2]. The clinical definition of PPH is excessive bleeding from the genital tract following the birth of the baby up to the end of the puerperium, which adversely affects the general condition of the patient evidenced by a rise in pulse rate and falling blood pressure. A 10% decline in post-partum hemoglobin concentration from antepartum levels is also another definition of PPH [3, 4]. PPH is classified as primary PPH, which is a common form of PPH that occurs within 24 h of delivery, and about 70% of primary PPH cases are due to uterine atony. Secondary (late) postpartum hemorrhage is defined as excessive bleeding occurring between 24 h after delivery of the baby and 6 weeks postpartum and most of the late PPH is due to retained products of conception infection, or both combined [1, 5].

Causes of PPH are related to abnormalities of one or more of four basic processes known as the four T’s; tone or abnormality of uterine contraction, tissue or retained products of conception, the trauma the of genital tract, and thrombin which is abnormalities with blood coagulation [6].

PPH is the leading cause of maternal death which is defined as the death of a woman while pregnant or within 42 days of termination of pregnancy, irrespective of the size and duration of the pregnancy, from pregnancy aggravated causes or its management, but not from accidental or incidental causes [7]. A woman could die within a few hours due to the absence of timely and proper intervention for postpartum hemorrhage [8]. Globally, nearly 830 women die every day from preventable causes related to pregnancy and childbirth of which 95% of all maternal deaths occur in low and lower-middle-income countries [9, 10]. The maternal mortality rate in Ethiopia is among the highest in the world of which PPH remains to be the leading cause [11].

Tremendous measures were taken to decrease maternal mortality. The WHO developed a set of guidelines for the prevention of PPH [2]. These guidelines are based on the best available evidence for different interventions for various components of active management of the third stage of labor. In Ethiopia, training was provided for health extension workers (HEWs) and traditional birth attendants (TBAs) which enables them to prevent PPH at the community level [11]. The Federal Ministry of Health of Ethiopia (FMOH) considered community-based distribution of misoprostol by Health Extension Workers and designed appropriate strategies to prevent PPH in rural Ethiopia [11]. In developing countries like Ethiopia, health systems face massive constraints that impede the delivery of emergency obstetric care, which is very helpful to save the lives of women who develop PPH. Once an event of PPH has occurred, the recommended management follows a sequential scheme of treatments that become increasingly intensive and invasive as the duration of hemorrhage advances [12]. In Ethiopia, the magnitude range from 3.06% in the study conducted the in Tigray region [13] to 32.8% a [14] in study conducted in the Amhara region. Factors that lead to increased risk of PPH include grand multiparity [15, 16], antepartum hemorrhage [16], cesarean section delivery [15, 16], pregnancy-induced hypertension [15–17], hydramnios and big baby [15, 17], prolonged and precipitate labor [18], mismanagement of third stage labor and previous PPH [19, 20], and lack of antenatal care (ANC) follow up [1, 3]. Even though different studies were conducted in Ethiopia the magnitude of PPH was not consistent and there was no national representative data. In addition independent studies that were published at the different setting of the country will not guide policy makes, clinicians and decision makers as the evidences were inconsistent and different. Therefore, this systematic review and meta-analysis aimed at estimating the magnitude of PPH and associated factors in Ethiopia.

## Methods

### Search strategy

This systematic review and meta-analysis were reported using Preferred Reporting Items for Systematic Reviews and Meta-Analyses (PRISMA) guideline [21]. The literature used for this review were identified through Medline, Pub Med, Cochrane library, the Web of Science, and Google Scholar search engines by developing search strategies. A Boolean operators such as AND, OR, Not were used with search terms such as “PPH”, “magnitude of PPH”, “prevalence of PPH”, “predictors”, “associated factors”, “postpartum women”, “maternal health care”, “Ethiopia”. Lists of retrieved articles were screened to make sure that all pertinent literature was included. Literature was downloaded to Endnote (version X7.2,) to maintain and manage citations, and facilitate the review process [22].

### Eligibility criteria

In this systematic review and meta-analysis, we included all studies that were conducted on the magnitude of PPH among women who gave birth in both public and private health facilities. The participants were women who gave birth at public and private facilities. We included all studies types that were published in the form of journal articles, master’s thesis, and dissertations in the English language. Both published and unpublished studies were included in the study. Articles, which were not fully accessed after at least two email contacts of the primary author were excluded. We excluded these articles because of the inability to assess the quality of articles in the absence of full text. No restriction was made for the date of publication and all studies conducted in Ethiopia were included.

### Outcome measurement

There were two main outcomes. The first outcome of interest was the magnitude of PPH, which was estimated as the total number of women with PPH cases divided by the total number of sample sizes multiplied by 100. From the primary study, PPH was operationalized as blood loss of more than 500 ml for vaginal delivery and ≥ 1000 ml for C/S or decrement in hematocrit value more than 10% from baseline value or maternal vital sign derangement (shock state). The second outcome was the factors associated with PPH, which were determined using the odds ratio (OR) and calculated based on binary outcomes from the included primary studies. The variables included in the review was the age of women (< 20 versus ≥ 20 years), ANC follow-up (Yes versus No), number of delivery (primipara versus multipara), previous history of PPH (Yes/No), and duration of labour (normal versus prolonged labour). ANC follow up was classified as ANC follow up or not (Yes/No). Postnatal Women were asked a history of ANC follow up for the current pregnancy. The variable was measured by binary variable “Yes” for those women who used one and above ANC visit and “No” for women who were not visited ANC during pregnancy.

### Data collection and quality assessment

The Joanna Briggs Institute (JBI) quality appraisal checklist for cross-sectional studies quality assessment tool was adapted and used to assess the quality of the included study [23]. Data were extracted by two data extractors (TT and MB) using a standardized data extraction checklist on Microsoft excel. Reference management software (Endnote version X7.2) was used to combine search results from databases and to remove duplicate articles initially. Then, studies were screened and excluded by their titles and abstracts. Full-text articles or reports were assessed for the remaining articles. Based on the predetermined inclusion and exclusion criteria, eligibility of the studies was evaluated. For the first outcome (magnitude of PPH), the data extraction checklist included author name, year of publication, region (an area where studies were conducted), study design, sample size, response rate and the number of participants with the PPH. For the second outcome (factors associated with PPH), data were extracted in a format of two by two tables, and then the log OR was computed based on the findings of the primary studies. Discrepancies between two independent reviewers were resolved by involving another reviewer (DM) after discussion for possible consensus. When articles did not have adequate data, corresponding authors of the research articles were contacted through their email.

### Data analysis and synthesis

The necessary information was extracted from each original study by using a format prepared in a Microsoft Excel spreadsheet. Then the data were exported to STATA for windows version 14 and used to calculate the pooled effect size with 95% confidence intervals. To check heterogeneity among the included studies, the Cochran *Q* test (Chi-squared statistic) and I^2^ statistic on forest plots were computed. Cochran’s *Q* statistical heterogeneity test is considered statistically significant at p ≤ 0.05. *I*^2^ statistics range from 0 to 100% and I^2^ statistic values of 0, 25, 50, and 75% were considered as no, low, moderate, and high degrees of heterogeneity, respectively [24]. A high degree of heterogeneity was observed for the first and second outcome, thus a random-effects model was used to determine the pooled magnitude of PPH. A meta regression was computed based on sample size and year of publication. In addition, a subgroup analysis was conducted to identify the source of potential random variation based on the region where the studies were conducted. Meta-regression was computed to see the presence of significant heterogeneity. A funnel plot was used to assess publication bias. Asymmetry of the funnel plot is an indicator of publication bias. In addition, Egger’s weighted regression and Begg’s tests were used to check publication bias [25]. Statistical significance of publication bias was declared at p-value of less than 0.05.

## Result

**Fig. 1:**
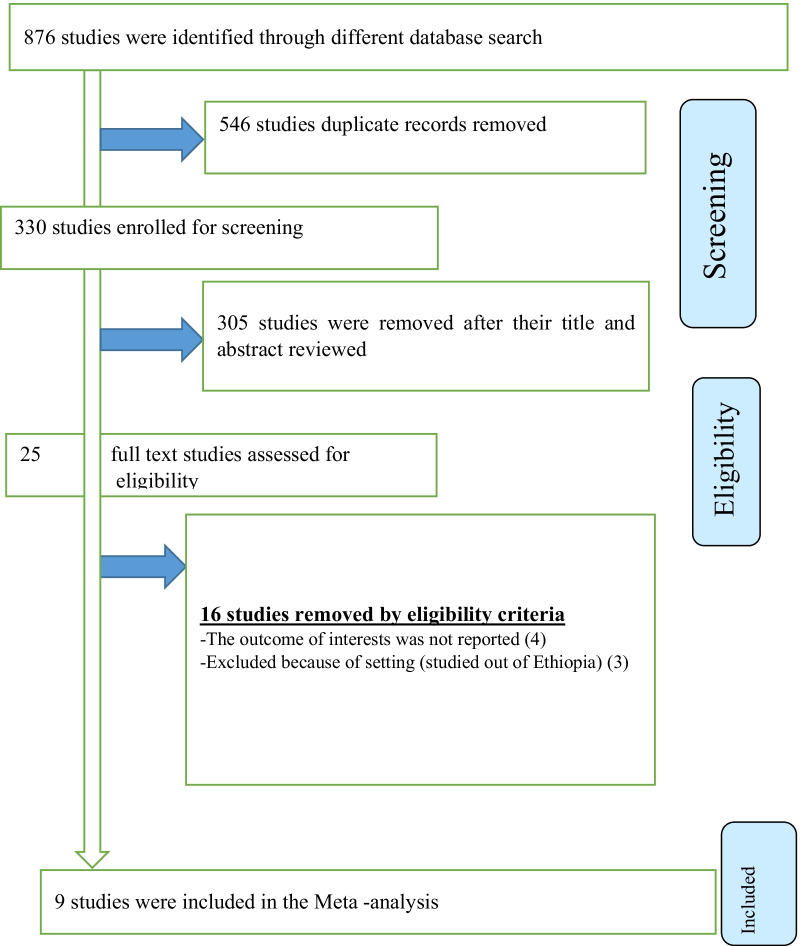
PRISMA flow diagram of included studies in the systematic review and meta-analysis of the magnitude of postpartum hemorrhage and associated factors in Ethiopia, 2020

A total of 876 studies were identified from various electronic databases and library catalogs. Of these studies, 546 articles records were identified and removed due to duplication. Reviewing titles and abstracts resulted in the exclusion of 305 irrelevant articles for our study. After assessing the full texts of the remaining articles, 16 studies were excluded as they did not meet the preset eligibility criteria. The remaining nine studies included in the final systematic review and meta-analysis (Fig. 1).

### Characteristics of included studies

All of the nine studies included in this study were published between 2014 to 2020 in peer review journals [13, 14, 26–31] and master’s thesis [32]. A total of 4032 study participants were included in the current systematic review and meta-analysis. The smallest sample size was 144 from a study conducted in the Amhara region, Debretabor Hospital [27], and the largest sample size was 1060 in a study conducted in Amhara region [31]. All included studies were cross-sectional in study design with 4 prospective [26–28, 30] and 5 retrospectives cross-sectional study design [13, 14, 29, 31, 32]. Regarding to study setting, five studies were conducted in the Amhara region [14, 27–29, 31], two studies in the Oromia region [30, 32], and two studies were from Tigrayi region [13] and SNNP [26]. The summary of the included articles is described in (Table 1).

**Table 1:** Summary of Included Studies on the magnitude of PPH in Ethiopia, 2019

S.N	Author	Year of publication	Region	Study area	Study design	Sample size	Quality score	Magnitude (95% CI)
1	Tesfaye et al. [30]	2018	Oromia	Bedelle	Prospective cross-sectional	200	9	9.50 (5.44, 13.56)
2	Mastewal [29]	2017	Amhara	Dessie	Retrospective cross-sectional	380	9	5.79 (3.44, 8.14)
3	Tatek et al. [28]	2014	Amhara	Gondar	Prospective cross-sectional	228	8	13.16 (8.77, 17.55)
4	Daniel et al.[27]	2019	Amhara	Debre tabor	Prospective cross-sectional	144	9	7.64 (3.330, 11.98)
5	Asmare et al. [14]	2018	Amhara	Debre markos	Retrospective cross-sectional	308	8	32.79 (27.55, 38.04)
6	Tariku [32]	2018	Oromia	Galamso	Retrospective cross-sectional	538	8	6.32 (4.26, 8.38)
7	Biruk, et al. [26]	2019	SNNP	Wachamo	Prospective cross-sectional	422	9	16.59 (13.04, 20.14)
8	Bewket et al. [31]	2020	Amhara	Bahirdar and Gondar	Retrospective cross-sectional	1060	9	8.87 (7.16, 10.58)
9	Mihret-ab et al. [13]	2020	Tigrayi	Mekelle	Retrospective cross-sectional	752	9	3.06 (1.83, 4.29)

*CI* confidence interval, *SNNP* Southern Nation Nationalities, and people

### Magnitude of PPH

**Fig. 2:**
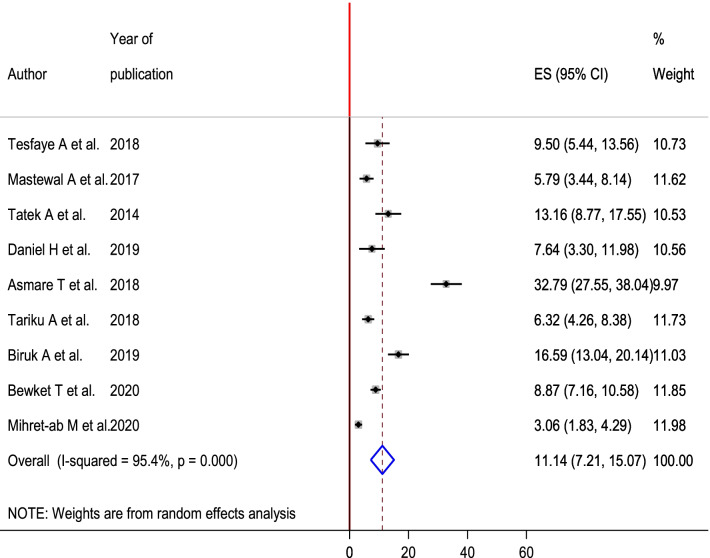
Forest plot of the pooled estimate of magnitude of postpartum hemorrhage in Ethiopia, 2020

High heterogeneity was observed across the included studies (I^2^ = 95.4, p < 0.001) and the random-effects model was used to estimate the pooled magnitude of PPH. The pooled magnitude of PPH was 11.14% (95% CI 7.21**,** 15.07). The highest (32.79% (95% CI 27.55, 38.04)) magnitude of PPH was observed in Debre Markos referral hospital, Amhara region [14] and the lowest 3.06% (95% CI 1.83, 4.29) magnitude of PPH was reported in Tigrayi region [13] (Fig. 2). To check for underlying heterogeneity, meta-regression models were done by using sample size and year of publication, but there was statistically insignificant for underlying heterogeneity (p = 0.785) and (p = 0.923), respectively (Table 2).

**Table 2:** Meta regression analysis based on sample size and year of publication

Variables	Coefficients	p-value
Publication year	0.0526758	0.923
Sample size	− 0.0013738	0.785

### Subgroup analysis and publication bias

**Fig. 3:**
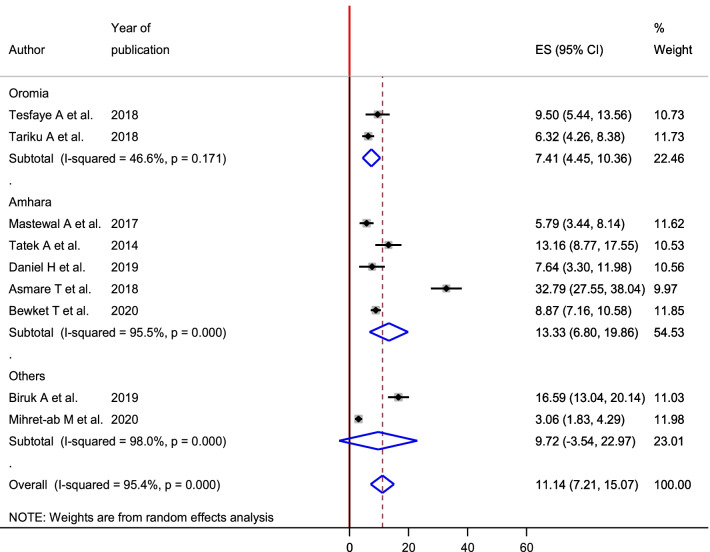
Sub group analysis of magnitude of postpartum hemorrhage among women gave birth in Ethiopia based on the region, 2020

**Fig. 4:**
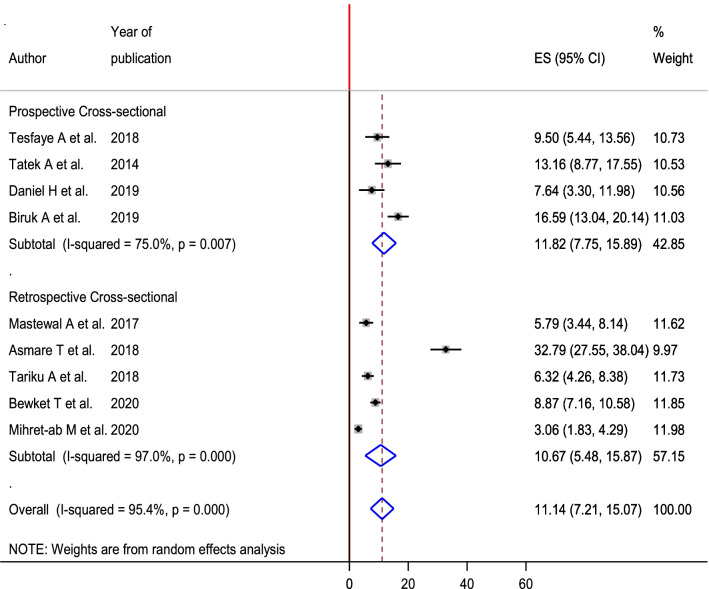
Sub group analysis of magnitude of postpartum hemorrhage among women gave birth in Ethiopia based on the timing of the study, 2020

To see heterogeneity among the included studies, subgroup analysis was executed based on the area where the studies were conducted and timing of study (prospective and retrospective study). Accordingly, the highest magnitude of PPH 13.32% (95% CI 6.80, 19.86) was observed in Amhara region and the lowest magnitude was observed in Oromia region with the magnitude of PPH 7.41% (95% CI 4.45, 10.36) (Fig. 3). According to timing of study, nearly the magnitude of PPH was equal among retrospective studies 10.67% (95% CI 5.47, 15.87) and prospective studies 11.81 (95% CI 7.75, 15.88) (Fig. 4).

**Fig. 5:**
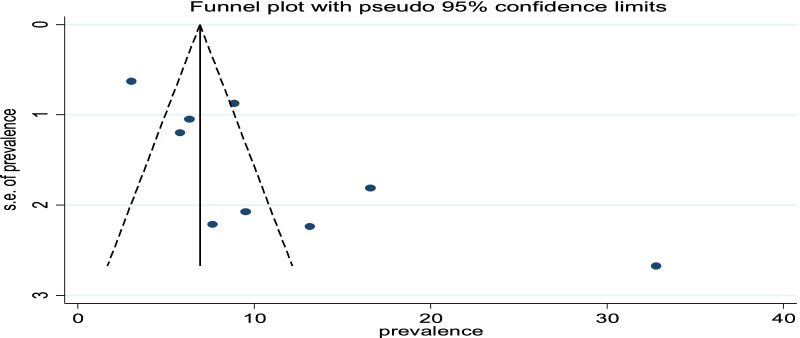
Funnel plot with 95% confidence limit of the magnitude of postpartum hemorrhage in Ethiopia, 2020

To see for the presence of publication bias, the graphical funnel plot and Egger’s test at a 5% significance level were executed. The visual examination of the funnel plot presented asymmetric which is an indicator for the presence of publication bias (Fig. 5). Egger’s test also showed significance presence of publication bias at a 5% significance level (p = 0.018).

**Fig. 6:**
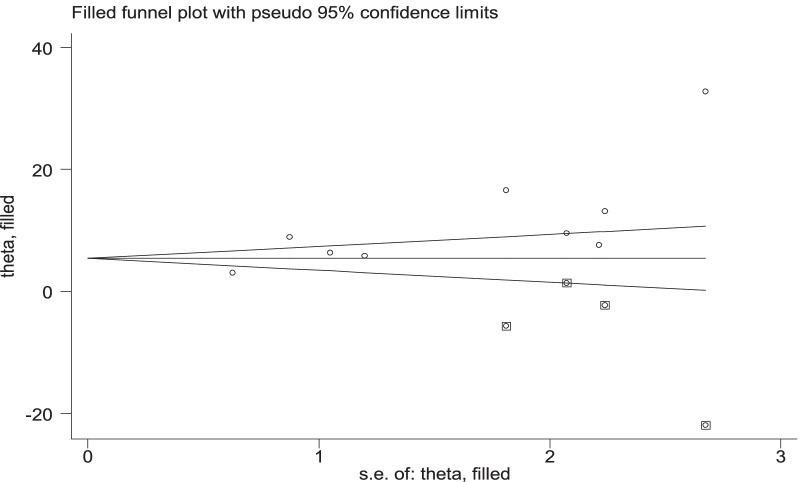
Result of trim and fill analysis for adjusting publication bias of the 9 studies, 2020

To reduce and adjust the publication bias in the studies, the trim and fill analysis was performed for estimation of the number of missing studies that might exist. Trim and fill analysis is a nonparametric methods for estimating the number of missing studies that might exist and it helps in reducing and adjusting publication bias in meta-analysis. In trim and fill analysis, four studies were imputed for missing studies and after adjustment for publication bias, the estimated pooled magnitude of PPH was 5.78 (95% CI 1.60, 9.95) (Fig. 6).

### Sensitivity analysis

**Fig. 7:**
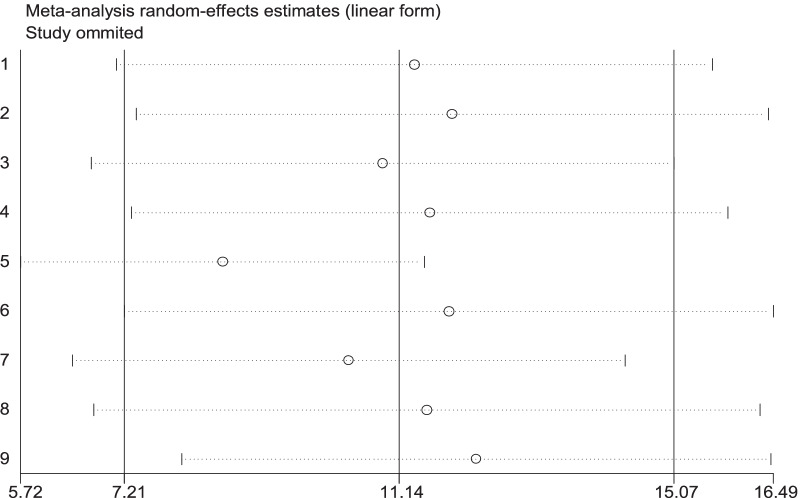
Result of sensitivity analysis of PPH in Ethiopia, 2020

Sensitivity analysis was computed to see any outliers and it showed there was no single study influence on overall included studies (Fig. 7).

### Factors associated with PPH

#### Association between age and PPH

**Fig. 8:**
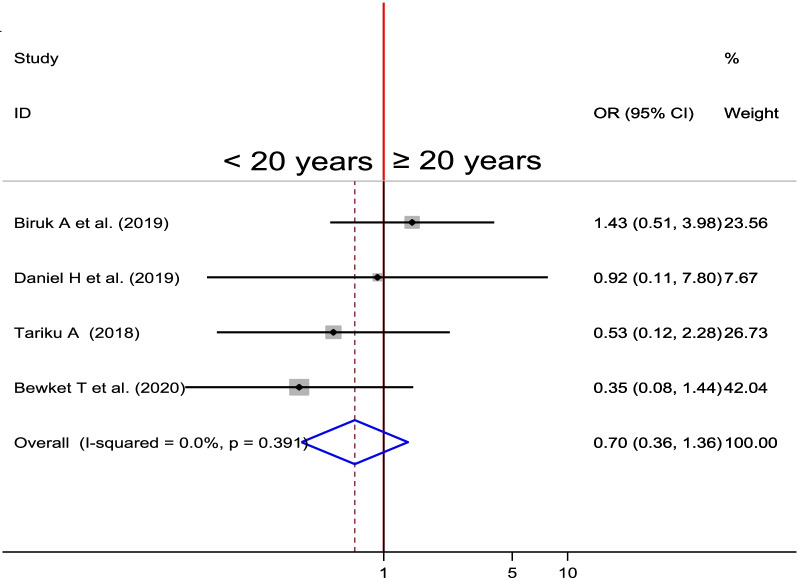
Forest plot of the pooled estimate of association between maternal age and PPH in Ethiopia, 2020

To examine the association between maternal age of mother and PPH, four studies were included in the analysis [26, 27, 31, 32]. A fixed-effects model was used to estimate the pooled association between age and occurrence of PPH (I^2^ = 0.00, P-value = 0.391). The pooled result of the analysis indicates that there was no statistically significant association between maternal age and development of PPH (OR = 0.70, CI 0.36, 1.36) (Fig. 8).

#### Association between PPH and ANC follow up

To identify the association between PPH and ANC follow up, three studies were included in the meta-analysis [27, 29, 31, 32]. To see their association, we found moderate heterogeneity across studies (I^2^ = 71.5%, p = 0.015), which is an indicator to use a random-effects model to estimate the pooled association between PPH and ANC follow up reported by the three studies with inverse variance.

**Fig. 9:**
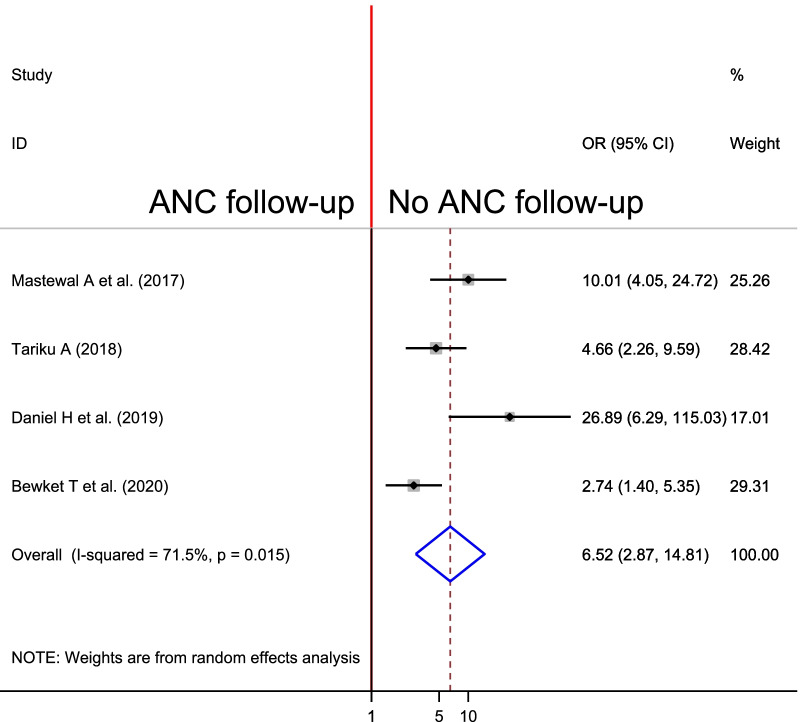
Forest plot of the pooled estimate of association between antenatal care follow up and PPH in Ethiopia, 2020

The pooled result of the analysis showed that there was a statistically significant association between PPH and ANC follow up. Accordingly, the likelihood of PPH was 6.52 times higher among women who had no ANC visits as compared to women who had ANC follow up. (OR = 6.52%, CI 2.87, 14.81) (Fig. 9).

### Association between number of delivery and PPH

**Fig. 10:**
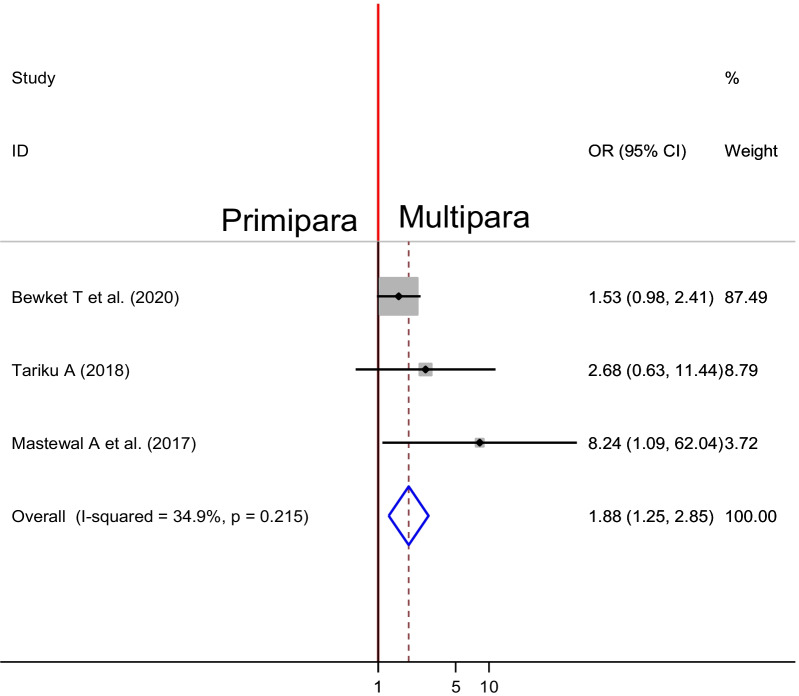
Forest plot of the pooled estimate of association between number of delivery and PPH in Ethiopia, 2020

Three studies were included to observe the association between number of pregnancy and PPH [29, 31, 32]. Accordingly, two of the included studies indicated no significant association between parity and PPH while, one study showed significant association. The pooled result showed there was there was significant association between parity and PPH, in which the odds of PPH was 1.88 times higher among multipara women than Primipara (OR = 1.88, 95% CI 1.25, 2.85) (Fig. 10).

#### Association between previous history of PPH and PPH

**Fig. 11:**
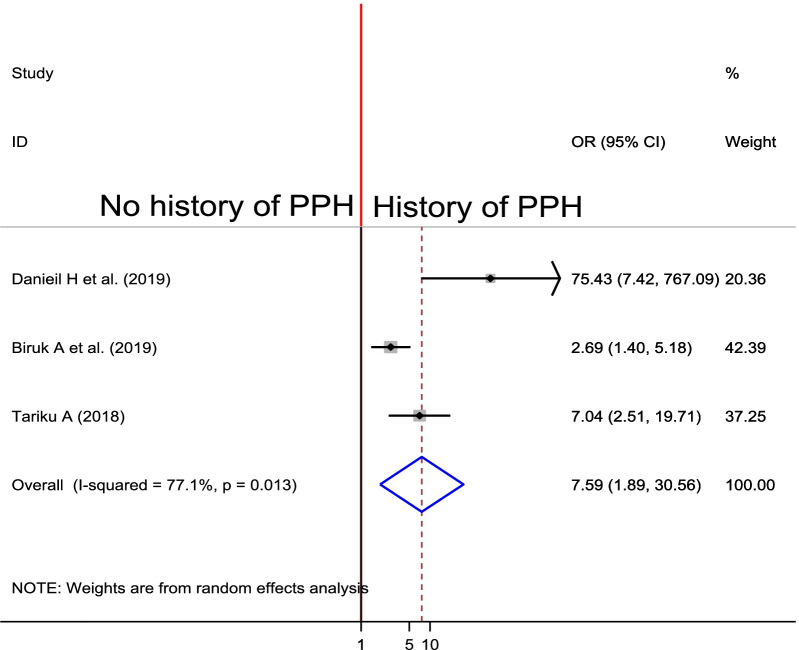
Forest plot of the pooled estimate of association between previous history of PPH and PPH in Ethiopia, 2020

To observe the association between previous history of PPH and PPH, three studies were selected for review [26, 27, 32]. All of the individual studies showed the significant association between previous history of PPH and the current occurrence of PPH. The pooled finding indicated the odds of developing PPH was 7.59 times higher among women who had previous history of PPH as compared to their counterparts (OR = 7.59, 95% CI 1.89, 30.56) (Fig. 11).

### Association between duration of labour and PPH

**Fig. 12:**
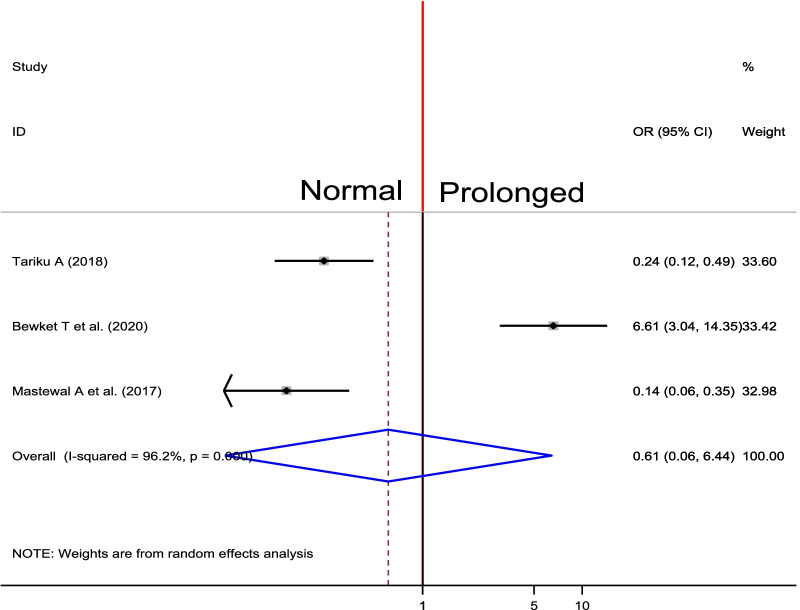
Forest plot of the pooled estimate of association between duration of labour and PPH in Ethiopia, 2020

Three studies were selected to the effect of duration of labour on occurrence of PPH [29, 31, 32]. In the primary studies, all of the included showed the significance association between duration of labour and PPH. The pooled estimates showed there was no significance association between duration of labour and occurrence of PPH (OR = 0.61, 95% CI 0.06, 6.44) (Fig. 12).

## Discussion

Globally, PPH is the primary cause of all maternal deaths and it is the leading cause of maternal mortality in low-income countries [9, 33]. This systematic review and meta-analysis, therefore, was conducted to assess the pooled magnitude of PPH and its association with ANC follow up among women gave birth in Ethiopia. Accordingly, the pooled magnitude of PPH in Ethiopia was 11.14% (95% CI 7.21**,** 15.07). This finding is comparable with a systematic and meta-analysis conducted globally, which reported the magnitude of PPH as 10.8% [34]. The finding is also comparable with the study conducted in Uganda with the magnitude of PPH 9.0% [15].

Conversely, the current pooled magnitude of PPH was higher than studies done Nigeria 1.13, 7.7%, and 2.72% [35–37]. Also, the finding was higher than studies conducted in Pakistan 7.24 [38], and Qatar which reported the magnitude of PPH as 5.1% [39]. The discrepancy might be due to differences in measurement of the outcome variable and improved ANC services. In previous studies, PPH was estimated by measuring blood loss in containers and gauze packs, while in the current review, most of the studies were used self-report of the patients for heavy bleeding and loss of consciousness to diagnose postpartum hemorrhage which may overestimate the number of cases. ANC is an important maternal health interventional area that reduces the occurrence of PPH by providing early diagnosis and treatment of preexisting disease, complications, and health promotion and disease prevention which are essential services to decrease pregnancy-related complications like PPH.

In the current systematic review and meta analysis, the pooled magnitude of PPH was lower than studies conducted among women who underwent delivery in Nigeria 25.6% [40] and Cameroon 23.6 [41]. This variation might be due to the study period and time frame to include women in the study after delivery. Most of the previous studies were conducted before 2015 which might be overestimating the number of patients with PPH due to a lack of accurate diagnosis of the cases. Besides, the previous studies included all women until their 42 weeks of post-delivery, while in the current review, most of the cases included in studies were all women who seek medical attention after 24 h to 6 weeks of delivery and this may decrease the number of patients with PPH.

Subgroup analysis was conducted to see the potential variation/heterogeneity among included studies. Accordingly, the lowest magnitude of PPH was observed in Oromia region and the highest magnitude of PPH was seen in Amhara region. This disimilarity might be due to the difference in the coverage of misoprostol to prevent PPH in different region and awareness of women towards the utilization of misoprostol. A previous study conducted in Ethiopia showed the coverage of Misoprostol utilization and awareness of women towards the utilization of Misoprostol is lower in Amhara region than Oromia region [42]. So the government, health professionals including health extension workers should work to increase the coverage of ANC visits by women during pregnancy inorder to decrease the number of PPH.

Women who had no ANC follow up were 6.52 more likely to develop PPH than women who had ANC follow up. This finding is in line with a study conducted in Nigeria in which inadequate ANC had a positive association with occurrence of PPH [43]. The justification for this is that women who booked ANC might be screened for risk factors of PPH and may get early diagnosis and treatment of preexisting disease, complications preparedness and prevention and this all reduces the occurrence of PPH.

The number of delivery is another factor which was significantly associated with occurrence of PPH. In this review, being multipara increase the odds of PPH and the finding is inline with studies conducted in Saudi Arabia in which PPH is more common in grand multipara [44–46]. This might be due to the impairment of uterus due to aging and scar, and as the number of delivery increase, the movement of blood towards the uterus might be changed due to impairment of uterine blood vessels. In addition, recurrent and over distention of uterus by multiple pregnancy increase the risk of uterine atony [47].

Having previous history of PPH was another factor in which women with previous history of PPH had sevenfold increased the odds of PPH in the subsequent pregnancy, and previous studies conducted in different countries supports this findings [46, 48–50]. Even though, the risk factors for recurrent occurrence of PPH is not well identified, having previous history of Oxytocin and maternal genetic makeup might be the risk factors for reccurent occurrence of PPH [46].

## Strength and limitation

This study has several strengths; such as various databases were used to search literature, and both published and unpublished studies were included in the study. The review was also not without limitations. First, the study included in the review was only published in the English language which may limit the number of studies that were reported in other languages. Second, in this review, only cross-sectional studies were included, which may decrease causal conclusions between PPH and associated factors. In addition, due to a small number of studies included in the final analysis (less than 10 studies), it is difficult to get the exact estimation of publication bias from the funnel plot.

## Conclusion

This systematic review and meta-analysis estimated the magnitude of PPH was high and this high magnitude of PPH was associated with lack of ANC follow up, multipara, and having previous history of PPH. Moreover, variation was also seen in different regions of the country. So, improving the ANC visits is recommended to decrease the PPH. All mothers need to be assessed carefully during the 24 h of post-delivery in the health institutions and health care providers need to strictly follow laboring mothers with partograph to detect and manage risk factors that cause PPH. In addition, all pregnant women need to receive health education and awareness to give birth in health institutions, and ANC follow up.

## Data Availability

The datasets analyzed during the current study are available from the corresponding author upon reasonable request.

## Abbreviations

ANC: Antenatal care
CI: Confidence interval
EDHS: Ethiopian Demographic and Health Survey
PPH: Postpartum hemorrhage
SSA: Sab Saharan Africa
SDG: Sustainable Development Goals
OR: Odd ratio
SNNP: Southern Nation Nationalities and People
WHO: World Health Organization
